# Effect of in‐hospital delays on surgical mortality for emergency general surgery conditions at a tertiary hospital in Malawi

**DOI:** 10.1002/bjs5.50152

**Published:** 2019-03-04

**Authors:** R. G. Maine, C. Kajombo, L. Purcell, J. R. Gallaher, T. D. Reid, A. G. Charles

**Affiliations:** ^1^ Department of Surgery University of North Carolina at Chapel Hill Chapel Hill North Carolina USA; ^2^ Department of Surgery Kamuzu Central Hospital Lilongwe Malawi

## Abstract

**Background:**

In sub‐Saharan Africa, surgical access is limited by an inadequate surgical workforce, lack of infrastructure and decreased care‐seeking by patients. Delays in treatment can result from delayed presentation (pre‐hospital), delays in transfer (intrafacility) or after arrival at the treating centre (in‐hospital delay; IHD). This study evaluated the effect of IHD on mortality among patients undergoing emergency general surgery and identified factors associated with IHD.

**Methods:**

Utilizing Malawi's Kamuzu Central Hospital Emergency General Surgery database, data were collected prospectively from September 2013 to November 2017. Included patients had a diagnosis considered to warrant urgent or emergency intervention for surgery. Bivariable analysis and Poisson regression modelling was done to determine the effect of IHD (more than 24 h) on mortality, and identify factors associated with IHD.

**Results:**

Of 764 included patients, 281 (36·8 per cent) had IHDs. After adjustment, IHD (relative risk (RR) 1·68, 95 per cent c.i. 1·01 to 2·78; *P* = 0·045), generalized peritonitis (RR 4·49, 1·69 to 11·95; *P* = 0·005) and gastrointestinal perforation (RR 3·73, 1·25 to 11·08; *P* = 0·018) were associated with a higher risk of mortality. Female sex (RR 1·33, 1·08 to 1·64; *P* = 0·007), obtaining any laboratory results (RR 1·58, 1·29 to 1·94; *P* < 0·001) and night‐time admission (RR 1·59, 1·32 to 1·90; *P* < 0·001) were associated with an increased risk of IHD after adjustment.

**Conclusion:**

IHDs were associated with increased mortality. Increased staffing levels and operating room availability at tertiary hospitals, especially at night, are needed.

## Introduction

Access to safe, timely and affordable surgical care is limited for over five billion people globally, most of whom live in low‐ and middle‐income countries (LMICs)^1^. Sub‐Saharan Africa bears a disproportionate burden compared with other regions, with approximately 35·7–99·4 per cent of the population unable to access surgical care^2^. Countries with low annual per capita healthcare expenditure (less than €350 per person) often prioritize maternal and child healthcare, and caesarean section alone can account for one‐third of all procedures in some countries^3^. The resulting deficit in general surgical and orthopaedic care creates a large burden of surgical disease.

Delays in surgical intervention for emergency conditions increase mortality^4^, ^5^, ^6^, ^7^, ^8^, ^9^, ^10^, ^11^, ^12^. The three primary sources of delay to surgical care for patients in limited‐resource settings include: delay in seeking care (pre‐hospital), delay in transferring to a facility equipped to perform the procedure (interfacility), and delay from presentation at a hospital able to provide definitive care to the start of the operative intervention (in‐hospital delays; IHDs). Numerous reasons for IHDs have been described in limited‐resource settings, including lack of human resources, infrastructure and equipment^1^, ^13^, ^14^. Few studies have assessed the impact of IHDs on perioperative mortality, or examined factors associated with IHDs.

Malawi is a landlocked country in south‐east Africa that can serve as a proxy for countries in the region. Access to surgical care is limited^15^, ^16^. The primary objective of the present study was to determine the effect of IHDs on in‐hospital mortality related to surgical interventions for emergency or urgent surgical conditions at a tertiary care centre. A secondary objective was to determine factors related to IHDs, in order to identify areas for future intervention. It was hypothesized that patients with emergency surgical conditions whose operations were delayed beyond 24 h after arrival in the hospital would have higher postoperative mortality than patients receiving earlier surgery.

## Methods

This analysis involved a cohort of patients collected prospectively in the Emergency General Surgery database who presented with abdominal symptoms to Kamuzu Central Hospital (KCH) in Lilongwe, Malawi. KCH is a 900‐bed tertiary hospital, acting as the referral centre for about six million people in the central region of Malawi. During the study period, surgical care was provided by four full‐time general surgeons, six surgical clinical officers, and 11 Malawian general surgery residents. KCH had four fully functional operating rooms staffed by one anaesthesiologist and six clinical officer anaesthetists. There were four adult ICU beds with ventilators. Available radiographic studies included plain and contrast radiography, abdominal ultrasonography and echocardiography. CT was available inconsistently.

All surgical patients, including children, who presented to the department as an emergency with abdominal pain from September 2013 to November 2017 were entered into a prospectively developed database. Data collected included basic demographics, presentation history, clinical characteristics, admission diagnosis, surgical interventions and outcomes. The database was maintained by data clerks and updated at KCH, with oversight from surgical residents and faculty.

Patients were included in the analysis if their surgical acuity was deemed an emergency or considered urgent based on admission diagnosis, determined by the admitting surgical team. These diagnoses included generalized peritonitis, acute appendicitis, bowel or gastric perforation, volvulus, non‐reducible hernia, and overt abdominal wound dehiscence. Peritonitis was based on clinical symptoms as assessed by the surgeon, reflecting both suspected visceral perforation and primary peritonitis, an entity seen in Malawi and other LMICs^17^. Patients managed without surgery, or whose surgery was delayed for more than 5 days, were excluded. In‐hospital delay to operative intervention was defined as a delay of more than 24 h from admission to operation. Diagnoses where a delay of more than 24 h was considered clinically appropriate were excluded.

The University of North Carolina institutional review board and the Malawi National Health Service review committee approved the study.

### Statistical analysis

Statistical characterization and comparisons were done with mean(s.d.) values, Student's *t* and Pearson's χ^2^ test as appropriate, using Stata® version 15.1 (StataCorp, College Station, Texas, USA). Bivariable and multivariable Poisson regression modelling were used to determine the relative risk (RR) of mortality for IHDs and other patient and clinical characteristics^18^. *A priori*, the factors included were: delayed surgery; patient characteristics (sex, age, co‐morbidities and pre‐hospital delay from symptom onset); and presentation and clinical characteristics (presentation year, abnormalities in initial vital signs (shock, defined as a shock index greater than 0·7 and tachypnoea), presenting diagnosis, admission ward). Similar analyses and multivariable modelling were applied to identify patient and presentation factors associated with IHD. Factors potentially associated with delay included: age, sex, transfer status, type of referring facility, days of pre‐hospital delay from onset, admission diagnosis, initial vital signs (shock and tachypnoea), presence of co‐morbidities, types of preoperative imaging, having any preoperative laboratory results, and weekend *versus* weekday presentation. Variables significant at *P* < 0·050 were included in the multivariable model.

## Results

**Figure 1:**
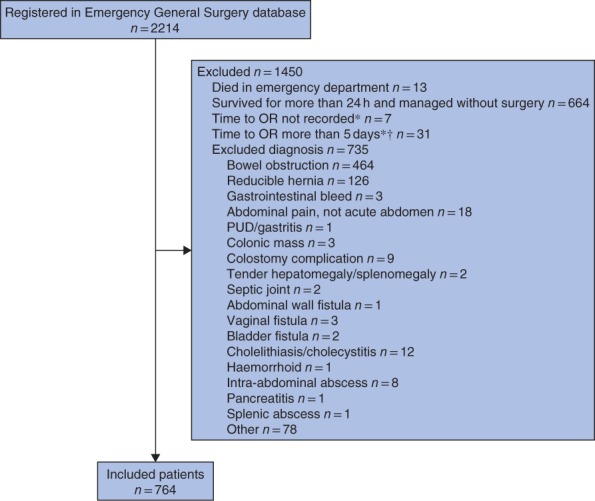
Inclusion of patients in the study. *For patients not excluded for other reasons. †Patients who waited for more than 5 days for the operating room (OR) were excluded as they were unlikely to have had an emergency condition; either misdiagnosis or clinical improvement was assumed to account for such a long delay. PUD, peptic ulcer disease

Over the study interval, the KCH acute care surgery database enrolled a total of 2214 patients, of whom 1450 did not meet study inclusion criteria (*Fig*. 1). Of the 764 included patients, 281 (36·8 per cent) experienced IHD (*Table* 1).

**Table 1:** Details of patients with in‐hospital surgical delay and timely surgical intervention

	All patients (*n* = 764)	Timely surgery (*n* = 483)	In‐hospital surgical delay (*n* = 281)	*P* ‡
Age (years)*	35·7(18·1)	35·3(17·8)	36·4(18·5)	0·212§
Sex ratio (F : M)	155 : 609	82 : 401	73 : 208	0·003
Transferred	646 of 762 (84·8)	415 of 482 (86·1)	231 of 280 (82·5)	0·182
Transferred from				0·187
Health centre	122 (18·9)	87 (21·0)	35 (15·2)	
District hospital	425 (65·8)	262 (63·1)	163 (70·6)	
Private hospital	85 (13·2)	58 (14·0)	27 (11·7)	
Tertiary hospital	14 (2·2)	8 (1·9)	6 (2·6)	
Pre‐hospital delay in presentation	*n* = 749	*n* = 474	*n* = 275	0·003
< 24 h	129 (17·2)	84 (17·7)	45 (16·4)	0·635
24–48 h	100 (13·4)	78 (16·5)	22 (8·0)	0·001
49–72 h	110 (14·7)	71 (15·0)	39 (14·2)	0·77
> 3 days	410 (54·7)	241 (50·8)	169 (61·5)	0·005
Weekend presentation	163 (21·3)	97 (20·1)	66 (23·5)	0·268
Night‐time admission	194 (25·4)	101 (20·9)	93 (33·1)	< 0·001
Any co‐morbidity†	94 of 720 (13·1)	67 of 450 (14·9)	27 of 270 (10·0)	0·063
Year of admission				< 0·001
2013	36 (4·7)	24 (5·0)	12 (4·3)	0·660
2014	250 (32·7)	171 (35·4)	79 (28·1)	0·038
2015	172 (22·5)	119 (24·6)	53 (18·9)	0·065
2016	138 (18·1)	87 (18·0)	51 (18·1)	0·962
2017	168 (22·0)	82 (17·0)	86 (30·6)	< 0·001
Occupation				0·008
Farmer	336 (44·0)	210 (43·6)	126 (44·8)	0·733
Other employment	218 (28·6)	144 (29·9)	74 (26·3)	0·296
Student/child	134 (17·6)	93 (19·3)	41 (14·6)	0·100
Unemployed	75 (9·8)	35 (7·3)	40 (14·2)	0·002

Values in parentheses are percentages unless indicated otherwise;

* values are mean(s.d.)

† Includes tuberculosis, history of recent treatment for malaria, hypertension, diabetes, known cancer and mental disability.

‡ χ^2^ test, except §Student's *t* test.

The mean age of all patients was 35·7(18·1) years, with no age difference between patients with IHD and those with a timely surgical intervention. Children under the age of 16 years represented 9·9 per cent (76 patients) of the total population. Women comprised 20·3 per cent (155 patients) of the entire cohort, but 26·0 per cent (73 women) of the IHD cohort (*P* = 0·003).

Most patients (646, 84·8 per cent) were transferred from another facility. Transfer rates did not differ between the IHD and timely intervention groups, nor did the type of transferring facility (*Table* 1). Weekday or weekend presentation was not associated with IHD (*P* = 0·268), neither was shock or tachypnoea on arrival (*Table* 2). Overall, only 13·1 per cent of patients had any co‐morbidity, which, again, was not associated with IHD (*Table* 1). The two most common diagnoses were generalized peritonitis (26·7 per cent) and incarcerated hernia (26·2 per cent) (*Table* 2). Admission diagnoses did not differ between patients subjected to IHD and those considered to have received timely intervention.

**Table 2:** Admission details for patients with timely surgery intervention and in‐hospital surgical delay

	All patients (*n* = 764)	Timely surgery (*n* = 483)	In‐hospital surgical delay (*n* = 281)	*P* †
Admission diagnosis				0·165
Acute abdomen/peritonitis	204 (26·7)	122 (25·3)	82 (29·2)	
Acute appendicitis	104 (13·6)	63 (13)	41 (14·6)	
Bowel or gastric perforation	109 (14·3)	66 (13·7)	43 (15·3)	
Volvulus	135 (17·7)	84 (17·4)	51 (18·1)	
Hernia, irreducible	200 (26·2)	142 (29·4)	58 (20·6)	
Other	12 (1·6)	6 (1·2)	6 (2·1)	
Admission ward	*n* = 763	*n* = 482	*n* = 281	0·604
To operating room	6 (0·8)	5 (1·0)	1 (0·4)	
Surgical ward	671 (87·9)	417 (86·5)	254 (90·4)	
Children's ward	58 (7·6)	40 (8·3)	18 (6·4)	
Other ward	5 (0·7)	4 (0·8)	1 (0·4)	
Intermediate care	14 (1·8)	9 (1·9)	5 (1·8)	
ICU	9 (1·2)	7 (1·5)	2 (0·7)	
Admission BP	*n* = 511	*n* = 313	*n* = 198	0·934
Hypotensive	21 (4·1)	13 (4·2)	8 (4·0)	
Normotensive	401 (78·5)	244 (78·0)	157 (79·3)	
Hypertensive	89 (17·4)	56 (17·9)	33 (16·7)	
Admission heart rate	*n* = 522	*n* = 322	*n* = 200	0·068
Bradycardia	21 (4·0)	18 (5·6)	3 (1·5)	
Normal	290 (55·6)	177 (55·0)	113 (56·5)	
Tachycardia	211 (40·4)	127 (39·4)	84 (42·0)	
Shock*	312 of 510 (61·2)	185 of 312 (59·3)	127 of 198 (64·1)	0·274
Admission respiratory rate	*n* = 445	*n* = 279	*n* = 166	0·735
Bradypnoea	2 (0·4)	1 (0·4)	1 (0·6)	
Normal	192 (43·1)	117 (41·9)	75 (45·2)	
Tachypnoea	251 (56·4)	161 (57·7)	90 (54·2)	

Values in parentheses are percentages.

* Defined as shock index (heart rate/systolic blood pressure) above 0·7.

† χ^2^ test.

Patients admitted at night (between 18·00 and 06·00 hours) experienced more IHDs than those with daytime admission (33·1 *versus* 20·9 per cent respectively; *P* < 0·001), and more patients in the IHD group were unemployed (14·2 *versus* 7·3 per cent; *P* = 0·002). The overall pre‐hospital delay from first symptoms to presentation at KCH differed between the two groups (*P* = 0·003). Delays before arrival at KCH were common; only 129 patients (17·2 per cent) presented within 24 h of the onset of symptoms (*Table* 1). In univariable regression analysis, patients presenting to KCH within 24–48 h of symptom onset were less likely to experience IHD (*P* = 0·001) than those who presented within 24 h of symptom onset, whereas patients who presented more than 3 days after symptoms started were more likely to experience IHD (*P* = 0·005). Patients who presented within 24 h of symptom onset were less likely to have shock (39·1 per cent *versus* 60·9 per cent in those with a later presentation; *P* < 0·001).

Most patients were admitted to the surgical ward (87·9 per cent) (*Table* 2). Only six patients (0·8 per cent) went directly to the operating room before admission to a ward, although this did not necessarily mean expedited surgical care. Admissions to intermediate care (1·8 per cent) and ICU (1·2 per cent) were rare. Admission location was not associated with IHD (*P* = 0·604).

Clinical and demographic factors associated with an increased risk of IHD are shown in *Table* 3. Univariable analysis indicated that female sex (RR 1·38, 95 per cent c.i. 1·13 to 1·68; *P* = 0·002), night‐time admission (RR 1·54, 1·29 to 1·85; *P* < 0·001), generalized peritonitis (RR 1·39, 1·05 to 1·82; *P* = 0·020) and time to obtain laboratory results at KCH (RR 1·60, 1·31 to 1·95; *P* < 0·001) were associated with a greater risk of delay. Duration of pre‐hospital delay, including delays in presentation and in transfer from a referring facility, were associated with IHD, with pre‐hospital delay of 24–48 h having a lower risk (RR 0·63, 0·41 to 0·98; *P* = 0·039). Except for generalized peritonitis, these factors remained significant in the multivariable model (*Table* 3), whereas patient age, type of referring facility, abnormal vital signs or shock on admission, year of admission, admission ward and preoperative imaging were not associated with IHD.

**Table 3:** Univariable and multivariable Poisson regression of factors associated with in‐hospital delay to surgery

	Univariable analysis		Multivariable analysis	
	Relative risk	*P*	Relative risk	*P*
Age category (years)				
0–10	0·93 (0·63, 1·37)	0·707		
11–20	0·73 (0·52, 1·01)	0·055		
21–40	1·00 (reference)			
41–60	0·91 (0·71, 1·15)	0·429		
> 60	1·11 (0·85, 1·46)	0·447		
Female sex	1·38 (1·13, 1·68)	0·002	1·33 (1·08, 1·64)	0·007
Transferred	0·85 (0·67, 1·07)	0·168		
Transferred from				
Health centre	1·00 (reference)			
District hospital	1·34 (0·99, 1·81)	0·062		
Private hospital	1·11 (0·73, 1·68)	0·634		
Tertiary hospital	1·49 (0·77, 2·91)	0·238		
Pre‐hospital delay in presentation				
< 24 h	1·00 (reference)		1·00 (reference)	
24–48 h	0·63 (0·41, 0·98)	0·039	0·63 (0·41, 0·96)	0·031
49–72 h	1·01 (0·72, 1·44)	0·927	0·93 (0·67, 1·30)	0·667
> 3 days	1·18 (0·91, 1·54)	0·213	1·07 (0·83, 1·38)	0·600
Shock	1·14 (0·90, 1·43)	0·279		
Initial respiratory rate				
Normal	1·00 (reference)			
Tachypnoea	0·92 (0·72, 1·17)	0·472		
Admission diagnosis				
Acute abdomen/peritonitis	1·39 (1·05, 1·82)	0·020	1·24 (0·94, 1·63)	0·134
Acute appendicitis	1·36 (0·98, 1·88)	0·062	1·37 (0·99, 1·89)	0·059
Bowel or gastric perforation	1·36 (0·99, 1·87)	0·058	1·19 (0·86, 1·63)	0·295
Volvulus	1·30 (0·96, 1·77)	0·091	1·29 (0·94, 1·76)	0·108
Hernia, irreducible	1·00 (reference)		1·00 (reference)	
Other	1·72 (0·94, 3·16)	0·078	1·45 (0·82, 2·59)	0·205
Year of admission				
2013	1·00 (reference)			
2014	0·95 (0·58, 1·56)	0·833		
2015	0·92 (0·55, 1·54)	0·764		
2016	1·11 (0·67, 1·85)	0·692		
2017	1·54 (0·94, 2·49)	0·083		
Weekend presentation	1·13 (0·91, 1·40)	0·259		
Night‐time admission	1·54 (1·29, 1·85)	< 0·001	1·59 (1·32, 1·90)	< 0·001
Admission ward				
To operating room	1·00 (reference)			
Surgical ward	2·27 (0·38, 13·60)	0·370		
Children's ward	1·86 (0·30. 11·60)	0·506		
Other ward	1·20 (0·10, 14·70)	0·887		
Intermediate care	2·14 (0·31, 14·70)	0·437		
ICU	1·33 (0·15, 11·60)	0·795		
Preoperative imaging*				
None	0·92 (0·75, 1·13)	0·415		
X‐ray	1·00 (0·83, 1·22)	0·982		
Ultrasound	1·22 (0·98, 1·52)	0·073		
CT	0·39 (0·06, 2·38)	0·305		
Time to obtain laboratory results	1·60 (1·31, 1·95)	< 0·001	1·58 (1·29, 1·94)	< 0·001

Values in parentheses are 95 per cent confidence intervals.

* Patients may have had more than one type of imaging study.

**Figure 2:**
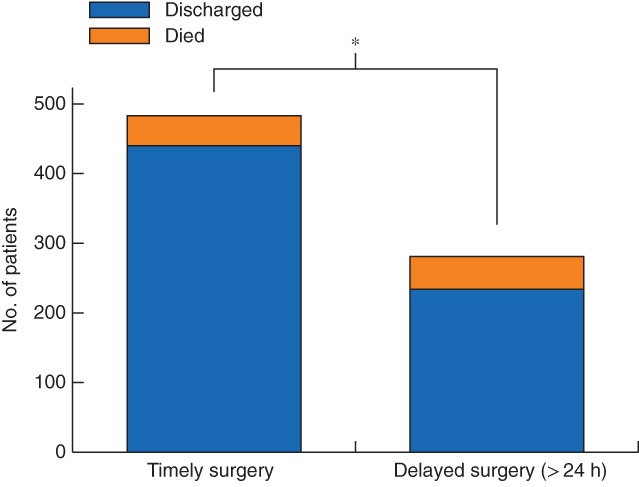
Mortality for timely and delayed emergency abdominal surgery in a central hospital in Malawi. *P = 0·001 (χ^2^ test)

The mortality rate for patients in the IHD group was higher than that for patients who had timely surgery: 47 of 281 (16·7 per cent) *versus* 43 of 483 (8·9 per cent) respectively (*P* = 0·001) (*Fig*. 2). Based on these mortality rates, a *post hoc* power calculation using the χ^2^ test demonstrated a power of 88·5 per cent.

In adjusted multivariable analysis, IHD of more than 24 h was associated with a relative mortality risk of 1·68 (95 per cent c.i. 1·01 to 2·78; *P* = 0·045) (*Table* 4). Although older age and a pre‐hospital delay of more than 3 days were associated with mortality in univariable analysis, in adjusted analysis only IHD and admission diagnoses (generalized peritonitis: RR 4·49, 1·69 to 11·95, *P* = 0·003; gastrointestinal perforation: RR 3·73, 1·25 to 11·08, *P* = 0·018) remained associated with an increased mortality risk.

**Table 4:** Univariable and multivariable Poisson regression of factors related to mortality

	Univariable analysis		Multivariable analysis	
	Relative risk	*P*	Relative risk	*P*
In‐hospital surgical delay	1·88 (1·28, 2·77)	0·001	1·68 (1·01, 2·78)	0·045
Age category (years)				
0–10	0·22 (0·03, 1·57)	0·131	0·00 (0·00, 0·00)	< 0·001
11–20	1·20 (0·64, 2·25)	0·577	1·01 (0·40, 2·50)	0·991
21–40	1·00 (reference)		1·00 (reference)	
41–60	1·68 (1·04, 2·73)	0·035	1·57 (0·85, 2·88)	0·151
> 60	2·37 (1·41, 3·99)	0·001	1·50 (0·71, 3·18)	0·293
Female sex	1·05 (0·65, 1·82)	0·836	0·68 (0·34, 1·38)	0·287
Pre‐hospital delay in presentation				
< 24 h	1·00 (reference)		1·00 (reference)	
24–48 h	1·15 (0·46, 2·86)	0·770	0·35 (0·08, 1·62)	0·180
49–72 h	1·43 (0·62, 3·33)	0·403	0·72 (0·25, 2·08)	0·548
> 3 days	2·13 (1·09, 4·17)	0·027	1·18 (0·54, 2·60)	0·681
Shock	1·43 (0·87, 2·34)	0·159	1·28 (0·71, 2·33)	0·416
Initial respiratory rate				
Normal	1·00 (reference)		1·00 (reference)	
Tachypnoea	0·70 (0·43, 1·13)	0·143	0·66 (0·40, 1·07)	0·090
Admission diagnosis				
Acute abdomen/peritonitis	6·54 (2·83, 15·10)	< 0·001	4·49 (1·69, 11·95)	0·003
Acute appendicitis	1·28 (0·37, 4·45)	0·695	1·35 (0·34, 5·42)	0·673
Bowel or gastric perforation	7·65 (3·23, 18·10)	< 0·001	3·73 (1·25, 11·08)	0·018
Volvulus	3·70 (1·47, 9·32)	0·005	2·64 (0·84, 8·26)	0·097
Hernia, irreducible	1·00 (reference)		1·00 (reference)	
Other	0·00 (0·0, 0·00)	< 0·001	0·00 (0·00, 0·00)	< 0·001
Any co‐morbidity	0·89 (0·48, 1·66)	0·709	0·74 (0·27, 2·02)	0·552

Values in parentheses are 95 per cent confidence intervals.

## Discussion

At a tertiary care facility in Malawi capable of delivering definitive surgical care for abdominal emergencies, 36·8 per cent of patients who met indications for emergency or urgent surgery on admission waited for longer than 24 h for their procedure. IHDs of more than 24 h for abdominal emergencies increased the risk of in‐hospital mortality by 67 per cent, compared with the risk in patients who received timely interventions (less than 24 h), after controlling for relevant co‐variables including pre‐hospital delay. Only female sex, night‐time admission and obtaining any laboratory results increased the risk of IHDs on adjusted analysis.

Existing data suggest that delays to surgical intervention are common in low‐resource settings, resulting in worse clinical outcomes. Timely operative intervention is a guiding surgical principle of acute abdominal emergencies^8^, ^12^, ^19^. The rate of IHDs of 36·8 per cent in the present study was similar to that in an earlier study^20^ at a well staffed teaching hospital in Nigeria, where 50 per cent of patients waited over 24 h, and 16 per cent waited more than 48 h for operative intervention. At a regional hospital in Uganda^21^ 48 per cent of 31 operations were delayed (median delay 14·8 h), and in the Ivory Coast^10^ 86 per cent of patients had a delay of more than 24 h from symptom onset to surgery, with more than 36 per cent waiting over 48 h. Delays were defined in the present study as longer than 24 h. Most surgeons would agree that this is too long to wait for emergency care, and several other studies, especially in LMICs, have used this definition^8^, ^11^, ^12^, ^22^, although some high‐income countries (HICs) have used shorter times^7^, ^8^, ^22^, ^23^. Future research to measure specific time intervals, including time to decision to operate, time to diagnostic studies and exact times to procedures, should clarify the length of an acceptable in‐hospital wait.

IHDs have been associated with worse outcomes in both HICs and LMICs^7^, ^10^. The overall mortality rate in the present study of 11·8 per cent was similar to rates of 10–19 per cent seen in other series^10^, ^15^, ^24^, ^25^ of abdominal operations in the region. In HICs, delays are often considered to have shorter intervals of less than 6 or 12 h^7^, ^8^, ^22^. Even these shorter delays are associated with increased mortality for emergency abdominal surgery^7^, ^8^, ^12^, as well as specific conditions including perforated diverticulitis^11^ and peptic ulcer disease^9^. In two Danish cohort studies^9^, ^12^, each hour of operative delay was associated with a 2 per cent reduction in survival. In sub‐Saharan Africa, limited data suggest that IHDs are also associated with worse outcomes for specific conditions. Patients with perforated peptic ulcer disease in the Ivory Coast had a hazard ratio for mortality of 15·6 if surgery was delayed more than 24 h from symptom onset^10^, and in patients with traumatic brain injury in Uganda IHDs increased mortality^26^.

Patients in LMICs encounter multiple types of delay, pre‐hospital, intrafacility and in‐hospital, that can affect mortality. Pre‐hospital delay was controlled for in this analysis to investigate the specific impact of in‐hospital delay on mortality. Interestingly, patients with 24–48 h of pre‐hospital delay were least likely to have IHD, whereas both shorter and longer pre‐hospital delays were associated with IHD. In a setting where long pre‐hospital delays are common, providers often observe patients and may not prioritize surgery for patients arriving with less than 1 day of symptoms. Similarly, when patients have a very long pre‐hospital delay, providers may assume less urgency because the patients have already tolerated their symptoms for several days. It is also possible that those with the greatest pre‐hospital delay, who had a higher rate of shock on arrival, experienced IHD as a result of the need for preoperative resuscitation. Much work in LMICs has focused on decreasing pre‐hospital delays by improving patient access to facilities where surgery can be performed. This study highlights that hospital arrival alone did not lead to rapid surgical intervention for many emergency surgical patients. Quality improvement efforts must identify and address the specific drivers of IHDs to decrease perioperative mortality in this population.

The reasons for delays in resource‐limited environments are context‐dependent. The Lancet Commission on Global Surgery proposed various reasons for IHD, including inadequate infrastructure and personnel, in addition to lack of consumables to address emergencies^1^, ^14^. Other studies have categorized delays as administrative, logistical or process‐based^13^. Although the present study found only female sex, night‐time presentation and obtaining laboratory results to be associated with IHD in adjusted analysis, it is acknowledged that the study was unable to consider several administrative and system factors. Availability of surgeons, supporting staff and operating room facilities, along with standards and speed of resuscitation, may all have been contributors to delay.

The disparities in delay to surgical care by sex have not been described previously. Female patients' abdominal symptoms may initially be diagnosed as gynaecological, delaying general surgical evaluation. Data regarding presurgical evaluation by other services were not available. Gender biases may influence providers' willingness to expedite surgical and anaesthetic care, decreasing the chances of timely intervention for women. Sociocultural norms may affect how women present, leading to downplaying of symptoms or providers attributing symptoms to ‘hysteria’.

IHDs were more common at night at KCH, a phenomenon also noted in Nigeria^20^. IHDs have consequences beyond increased mortality for emergency surgical patients admitted at night, as the backlog that develops can compound delays the following day for surgical services. Increasing operating room availability and staffing, including an on‐call or backup team to staff additional operating theatres, and improved access to equipment could significantly reduce IHDs associated with night‐time admissions.

Studies on risk of IHD to surgery in both low‐ and high‐resource settings have identified patient‐related constraints^14^. In Nigeria, financial constraints and delays for investigations were considered important factors leading to delays for abdominal emergencies^20^. In that healthcare system, patients must provide their own antibiotics and intravenous fluids. Although the role of patient resources in IHDs at KCH was not evaluated specifically, available supplies at KCH were provided free to patients, so this element was unlikely to have delayed operations significantly. Conversely, limited access to transport, shortages or malfunctions in key equipment, including sterilization and operative supplies, were not uncommon at KCH, although the extent of their contribution to delays was not evaluated.

Several studies^7^, ^8^, ^13^, ^20^, ^26^, ^27^ have noted delays associated with obtaining diagnostic test results. Preoperative imaging was not associated with IHDs in this cohort, but the minority of patients who had preoperative laboratory assessments did experience more delay. Although haemoglobin results were usually available within a few hours, biochemistry results could be delayed for days at KCH. The reason why obtaining laboratory work delayed surgery remains unclear.

A number of limitations reflect the design of the present study. In selecting only surgical patients with recorded times to intervention for whom the admission diagnosis was consistent with the need for urgent surgery, a specific cohort of patients were analysed from the registry. It is possible that some patients were thought to have improved after initial evaluation, resulting in longer times to intervention. The study may have underestimated the impact of IHDs on mortality, as patients who never made it to the operating room were excluded, as were those delayed as a result of initial misdiagnosis and patients for whom exact times from admission or decision to operate to surgery were not recorded.

Surgical delays for emergency general surgery conditions were common in this population in Malawi, with over one‐third of patients who experienced delays having a significantly higher risk of mortality. Quality improvement work focused on IHDs could decrease mortality in sub‐Saharan Africa. These efforts should focus on improving delivery of surgical care, not just at central hospitals but across all levels of the healthcare system, including district hospitals, to decrease in‐hospital and other sources of patient delay.
